# The Outcome of Pregnancy Among Tobacco Users in Tertiary Care Hospital of Chhattisgarh Province of India

**DOI:** 10.7759/cureus.32877

**Published:** 2022-12-23

**Authors:** Tripti Nagaria, Neha Singh, Himani Punshi, Monika Dengani, Shailendra Agrawal, Kamlesh Jain, Nirmal Verma, Smrity Naik

**Affiliations:** 1 Department of Obstetrics and Gynecology, Pt. Jawahar Lal Nehru Memorial (JNM) Medical College, Raipur, IND; 2 Department of Microbiology, Pt. Jawahar Lal Nehru Memorial (JNM) Medical College, Raipur, IND; 3 Department of Community Medicine, Pt. Jawahar Lal Nehru Memorial (JNM) Medical College, Raipur, IND

**Keywords:** tobacco adverse effects, tobacco users, fetal outcome, pregnancy outcome, pregnancy

## Abstract

Background: Tobacco use is responsible for numerous adverse pregnancy outcomes for females and their infants. The aim of this study was to explore the adverse outcome of tobacco use among pregnant females.

Method: A cross-sectional study was conducted on 1250 females in the third trimester of pregnancy from April to June 2022, which were exposed to tobacco use in the form of gudaku, tobacco chewing, gutka, or smoking. Complications and outcomes during and after pregnancy were recorded based on self-administered questionnaires. Statistical analysis was performed using the Statistical Package for Social Sciences (SPSS) (IBM SPSS Statistics, Armonk, NY) software version 20.0 for categorical data, frequencies (n) and percentages (%) were calculated, and the chi-square test was used for determining intergroup differences.

Results: Out of 1250 females, tobacco exposure was present among 429 (34.3%), and 821 (65.7%) had no tobacco exposure. Of 429, 36.10% of females complained about complications such as abortion (1.60%), antepartum hemorrhage (0.90%), congenital anomaly (0.20%), infertility (1.20%), intrauterine fetal death (IUFD) (0.50%), intrauterine growth restriction (IUGR) (0.90%), oligohydramnios (OLIGO) (3.30%), preterm labor (18.40%), premature rupture of membrane (6.30%), and anemia (2.80%), which were slightly higher than the females with no tobacco exposure. In tobacco users, obstructive complications were found to be significant with a p value of 0.0036.

Conclusion: Our study concluded that tobacco use could have an adverse effect on their fetus and infants, as well as the pregnant females themselves. Policymakers need to ensure effective strategies that pregnant females, their partners, and close relatives need to have enough knowledge to avoid potential risks.

## Introduction

Smoking has assorted adverse effects on pregnant females and their developing fetuses, including a variety of squeals that will stay with the fetus for the rest of her life. Smoking in any form creates risks and adverse effects on maternal and neonatal outcomes [1]. Smoking during pregnancy, whether actively or passively, increases the chance of spontaneous abortion, placenta previa (when the placenta partially or fully blocks the internal cervical os), and placental abruption (premature separation of the placenta from the uterine wall). Additionally, smoking cigarettes that contain tobacco increases the risk of premature membrane rupture, placenta previa, and preterm delivery [2]. Numerous studies carried out in various nations have demonstrated a significant inverse relationship between maternal smoking during pregnancy and birth weight or significant positive associations between maternal smoking during pregnancy and the risk of low birth weight (LBW), preterm birth, and small-for-gestational-age (SGA) birth [3-6]. On the other hand, the impact of a pregnant female who smokes passively is uncertain and has not been thoroughly researched [5-7]. Since the person who inhales the smoke frequently has no choice, passive smoking is also known as "involuntary smoking."

Smoking during pregnancy, whether actively or passively, has long been regarded as a significant risk factor for intrauterine development retardation [8]. There is strong epidemiological evidence that smoking during pregnancy increases the incidence of placenta previa, abruption of the placenta, early rupture of membranes, and preterm delivery [9]. In addition to other negative consequences, maternal smoking was found to be strongly related to preterm delivery; intrauterine growth retardation; a small head circumference; a low appearance, pulse, grimace, activity, and respiration (APGAR) score at five minutes and stillbirths; and neonatal deaths, in a Swedish study of females who gave birth between 1983 and 1996 [10]. Despite the fact that environmental tobacco smoke (ETS) exposure was not significantly correlated with the risk of low birth weight (LBW) or preterm delivery, a study found a significant association between ETS exposure and lower mean birth weight (weighted mean difference: 60 g; 95% confidence interval {CI}: 89-39 g) [11]. It is a known fact that the primary preventable cause of death and disease among people is tobacco use. According to the World Health Organization, smoking contributes to an estimated five million premature deaths during pregnancy worldwide in each year [12]. Chhattisgarh has the highest consumption of tobacco across the country with 39.1% of people in the state using tobacco, and it is believed that in this state, the female ratio of tobacco users is not less than that of males. Hence, the present study was designed to investigate further the effects of tobacco use in pregnant females and adverse pregnancy outcomes at the primary level on pregnant females admitted to tertiary care in Dr. Bhimrao Ambedkar Memorial Hospital, Raipur, Chhattisgarh. The outcome variables included obstetric complications such as abortion, antepartum hemorrhage, congenital anomaly, infertility, intrauterine fetal death (IUFD), intrauterine growth restriction (IUGR), oligohydramnios (OLIGO), premature rapture of membrane (PROM), anemia, mode of delivery, fetal weight (kg), APGAR score, and neonatal intensive care unit (NICU) admission.

## Materials and methods

A cross-sectional study was conducted at the Department of Obstetrics and Gynecology in Dr. BRAM Hospital, Raipur, Chhattisgarh, India. The inclusion criteria for the study group consisted of all pregnant females who were getting admitted for delivery between the ages of 20 and 40 years with singleton pregnancies and having no chronic illnesses such as diabetes, arthritis, Alzheimer's disease, cancer, and chronic obstructive pulmonary disease (COPD). Antenatal patients with a history of major medical illnesses and surgery in the recent past year; with known cases of neurological and mental illnesses (anxiety disorders, epilepsy, depression, post-traumatic stress disorder {PTSD}, etc.); on medications, e.g., benzodiazepines, amphetamines, opioids, or any neuroleptics; and with addiction to alcohol were excluded from the study. A total of 1250 females were admitted for delivery who were interviewed for tobacco use based on the predesigned pro forma.

The sample size was calculated using Cochran’s formula. The z-value is 1.96 at 95% confidence interval, the proportion of tobacco consumption among pregnant females is 0.62, and the desired level of precision is 5%. Females were enrolled with the gestational age taken between 28 and 37 weeks at delivery. The study period for recruitment was from April to June 2022. The study approval was obtained from the Institutional Ethics Committee (IEC) of Pt. Jawahar Lal Nehru Memorial (JNM) Medical College (No./MC/Ethics/2022/178). Informed written consent was obtained from each of the pregnant females before taking an interview to enroll in the study. Pregnant females were interviewed based on a predesigned questionnaire at the time of delivery, and background characteristics such as educational status, age group, occupation, diet, tobacco use, period of using tobacco (in years), and gestation age were recorded. Active tobacco users included in the study were the females who used tobacco in the form of gudaku, tobacco chewing, gutka, and smoking. Passive users were considered females who breathe in secondhand smoke regularly as a result of having a smoker at home or at the workplace. The period of tobacco exposure included was from less than one year to more than 10 years. Abortion, antepartum hemorrhage, congenital anomaly, infertility, IUFD, IUGR, OLIGO, preterm labor, PROM, anemia, mode of delivery such as normal vaginal delivery (NVD), preterm vaginal delivery (PTVD), vaginal-induced delivery (VID), fetal weight (kg), APGAR score at one minute and five minutes, NICU admission needed or not for the newborn were recorded as outcome measures of obstetric complications. Maternal anemia, defined as hemoglobin of <11 g/dL at the time of delivery, was also recorded. For categorical data, frequencies (n) and percentages (%) were calculated using the statistical analysis program Statistical Package for Social Sciences (SPSS) (IBM SPSS Statistics, Armonk, NY) software version 20.0, and the chi-square test was used to identity the association between the categorical variables.

## Results

Pregnant females admitted to the hospital were interviewed before delivery based on pre-designed and self-administered questionnaires. Based on their background characteristics (Table 1)

**Table 1:** Background characteristics of pregnant females and newborn APGAR: appearance, pulse, grimace, activity, and respiration; LSCS: lower segment cesarian section; NICU: neonatal intensive care unit; NVD: normal vaginal delivery; PTVD: preterm vaginal delivery; VEG: vegetarian; VID: vaginal-induced delivery

Background characteristics		Frequency	Percentage
Educational status	Illiterate	105	8.4
	Primary	201	16.1
	Secondary	650	52
	Higher secondary	197	15.8
	Graduate	97	7.8
Age (years)	≤20	94	7.5
	21-25	722	57.8
	26-30	380	30.4
	31-35	50	4
	36-40	4	0.3
Occupation	Housewife	1249	99.9
	Private job	1	0.1
Diet	Mixed	1078	86.2
	VEG	172	13.8
Tobacco users (gudaku, tobacco chewing, gutka, or smoking)	No	821	65.7
	Yes	429	34.3
Gestational age	Less than 28	25	2
	28-32	50	4
	33-37	216	17.3
	More than 37	959	76.7
Obstetric complication	Absent	819	65.5
	Present	431	34.5
Mode of delivery	Abortion	20	1.6
	LSCS	535	42.8
	NVD	685	54.8
	PTVD	1	0.1
	VID	9	0.7
Sex (baby)	Female	593	47.4
	Male	637	51
	Not known (aborted)	20	1.6
Newborn weight (kg)	<1.5	56	4.5
	1.5-2.5	313	25
	2.5-3.5	867	69.4
	>3.5	14	1.1
APGAR score at the first minute	Less than 7	857	68.6
	7 or more	393	31.4
APGAR score at the fifth minute	Less than 7	231	18.5
	7 or more	1019	81.5
NICU Admission	Need resuscitation	16	1.3
	NICU admission	40	3.2
	No	1194	95.5
	Total	1250	100

Educational status was recorded as secondary school (52%), illiterate (8.4%), graduation (7.8%), primary (16.1%), and higher secondary (15.8%). Most of the pregnant females belonged to the age group of 21-25 years (57.8%) and 26-30 years (30.4%); however, very few, only four females, in the age group of 36-40 years (0.3%) participated in the study. Of 1250, 1249 (99.9%) were homemakers, whereas only one (0.1%) was a private professional; 1078 (86.2%) were from a mixed diet, and 172 (13.8%) were vegetarian; out of 1250, 429 (34.3%) were tobacco users, and 821 (65.7%) were non-users. Most of the females, 959 (76.7%), admitted to the hospital were from more than 37 weeks, whereas only 25 (2%) were from less than 28 weeks. Overall, obstetric complications were found in 431 (34.5%) pregnant females out of 1250. Natural vaginal delivery (NVD) happened in 54.8% of females and lower segment cesarian section (LSCS) in 42.8%. A total of 637 (52%) male babies and 593 (47.4%) female babies were born from the females admitted to the hospital, whereas 20 (1.6%) newborns got aborted; 867 (69.4%) newborns had birth weights of 2.5-3.5 kg, and 56 (4.5%) and 14 (1.1%) babies had birth weights of more than 3.5 kg and less than 1.5 kg, respectively; 1.3% and 3.2% of babies needed NICU resuscitation and NICU admission, respectively, while 95.5% of babies did not need any NICU admission (Table 2).

**Table 2:** Obstetric complications present among pregnant females IUFD: intrauterine fetal death; IUGR: intrauterine growth restriction; OLIGO: oligohydramnios; PROM: premature rapture of membrane

Obstetric complication	Tobacco non-users (n=821)		Tobacco users (n=429)	
	Number	%	Number	%
No complication	590	71.90	274	63.90
Abortion	8	1.00	7	1.60
Antepartum hemorrhage	10	1.20	4	0.90
Congenital anomaly	1	0.10	1	0.20
Infertility	6	0.70	5	1.20
IUFD	6	0.70	2	0.50
IUGR	11	1.30	4	0.90
Low amniotic fluid (OLIGO)	24	2.90	14	3.30
Preterm labor	117	14.30	79	18.40
PROM	26	3.20	27	6.30
Anemia	22	2.70	12	2.80
Total	821	100.00	429	100.00

Table 3 depicted obstetric complications during pregnancy in tobacco-exposed and nonexposed pregnant females; it was found that complications were slightly higher in the females who were consuming tobacco. Females who use tobacco were found preterm with other complications, PROM, low amniotic fluid, and infertility, 18.40%, 3.20%, 3.30%, and 1.20% respectively, which were slightly higher as compared to non-users (14.30%, 3.20%, 2.90%, and 0.70%, respectively).

**Table 3:** Association of tobacco users and non-users with maternal and neonatal outcome APGAR: appearance, pulse, grimace, activity, and respiration; df: degrees of freedom; LSCS: lower segment cesarian section; NICU: neonatal intensive care unit; NS: not significant; NVD: normal vaginal delivery; S: significant; PTVD: preterm vaginal delivery; VID: vaginal-induced delivery

Maternal and child characteristics		Tobacco exposure						
		Absent (n=821)		Present (n=429)		Chi-square	df	P value
		Number	%	Number	%			
Educational status	Graduation	64	7.80	33	7.70	1.739	4	0.784 (NS)
	Higher secondary	122	14.90	75	17.50			
	Illiterate	72	8.80	33	7.70			
	Primary	134	16.30	67	15.60			
	Secondary	429	52.30	221	51.50			
Age group	≤20	65	7.90	29	6.80	4.768	4	0.312 (NS)
	21-25	476	58.00	246	57.30			
	26-30	250	30.50	130	30.30			
	31-35	29	3.50	21	4.90			
	36-40	1	0.10	3	0.70			
Gestational age at the time of delivery	28-32	29	3.50	21	4.90	5.081	3	0.166 (NS)
	33-37	142	17.30	74	17.20			
	More than 37	638	77.70	321	74.80			
	Less than 28	12	1.50	13	3.00			
Obstetric complication	Absent	590	71.86	274	63.86	8.436	1	0.0036 (S)
	Present	231	28.13	155	36.13			
Mode of delivery	Abortion	10	1.20	10	2.30	4.212	4	0.378 (NS)
	LSCS	360	43.80	175	40.80			
	NVD	443	54.00	242	56.40			
	PTVD	1	0.10	0	0.00			
	VID	7	0.90	2	0.50			
Newborn weight	<1.5	32	3.90	24	5.60	2.001	3	0.572 (NS)
	>3.5	9	1.10	5	1.20			
	1.5-2.5	209	25.50	104	24.20			
	2.5-3.5	571	69.50	296	69.00			
APGAR score at the first minute	7 or more	269	32.80	124	28.90	1.948	1	0.163 (NS)
	Less than 7	552	67.20	305	71.10			
APGAR score at the fifth minute	7 or more	690	84.00	329	76.70	10.115	1	0.001 (S)
	Less than 7	131	16.00	100	23.30			
NICU admission	Need resuscitation	8	1.00	8	1.90	2.403	2	0.301 (NS)
	NICU admission	24	2.90	16	3.70			
	No	789	96.10	405	94.40			
Total		821	100.00	429	100.00			

A significant association was found between the use of tobacco with obstetric complications and an APGAR score at five minutes with an odds ratio of 1.43 and 1.60, respectively.

## Discussion

The current status of Indian tobacco control strategies appears to be supported by the findings and suggestions from international studies. However, India has a far wider range of tobacco and health issues, necessitating the development of plans with the aid of regional studies on tobacco control. Indian females don't traditionally smoke or use tobacco, but this perception is changing recently with time [13]. Smoking or tobacco use especially during pregnancy has been firmly shown to have a number of negative impacts on the fetus. Global Adult Tobacco Survey (GATS) reveals that in a span of five years, tobacco use in females increased at a higher rate of 25.2%-41.6% from the years 2005-2006 to 2009-2010 [14]. According to our present findings, pregnant females with tobacco exposure complained about obstructive complications such as abortion (1.60%), antepartum hemorrhage (0.90%), congenital anomaly (0.20%), infertility (1.20%), intrauterine fetal death (0.50%), intrauterine growth restriction (0.90%), oligohydramnios (3.30%), preterm labor (18.40%), premature rupture of membrane (6.30%), and anemia (2.80%). Obstructive complications were found to be significant with a p value of 0.0036 and APGAR score at the fifth minute with a p value of 0.001. Likewise, other research findings also indicate that tobacco use in the form of smoking or smokeless while pregnant reduces the birth weight of an unborn baby, shortens gestational age, and raises the risk of stillbirth [15,16].

Smoking during pregnancy has indeed been linked to premature membrane rupture, abruptio placentae, placenta previa, preterm birth, intrauterine growth restriction, and sudden infant death syndrome. According to some research, smoking causes 15% of premature births, 20%-30% of low birth weight of babies, and a 15% increase in overall perinatal death. One of the most significant risk factors linked to poor perinatal outcomes is cigarette smoking [17]. Research also showed that prenatal exposure to tobacco increases the incidence of respiratory infections, allergies, asthma, and pediatric cancer, as well as has neurobehavioral effects on the children's health in the long term. It has been discovered that the high quantity of tobacco use during pregnancy increases the risk of the majority of these illnesses [18]. The findings from a multiple linear regression analysis also showed the effects of maternal smoking on birth outcomes that newborns that were exposed to nicotine (through urine cotinine levels higher than 5 g/ml) during pregnancy experienced a birth weight reduction of around 100 g than the normal range [19]. In nearly one-third of maternal and cord blood samples from the tobacco-exposed group, nicotine absorption was recorded in investigations. Because the liver enzyme CYP2A6 is activated during pregnancy, the metabolism of nicotine and cotinine increases by 60% and 40%, respectively [20].

Some biochemical factors have been proposed; however, the exact mechanism by which prenatal exposure to cigarette toxins predisposes to stillbirth is not yet fully understood. A substantial risk factor for stillbirth is fetal hypoxia, which is caused when carbon monoxide from tobacco smoking binds to hemoglobin and creates carboxyhemoglobin in both maternal and fetal blood [21,22]. Nicotine has been demonstrated to have negative direct effects on a number of key processes in the development of the placenta, including a decrease in placental angiogenic factors and an inhibitory effect on trophoblast invasion [23], which can result in both direct placental complications and fetal growth restriction [24]. Females who are passionate may be more prone to smoke and have impulsive children, or prenatal tobacco exposure may result in more impulsive offspring. Research from animal experimental research proves particular CNS changes brought on by prenatal nicotine exposure that may have an impact on the behavior in offspring. As a result, the observed adverse results are most probably triggered by both genetic and teratological etiologies [25]. It has been also reported somewhere that smokeless tobacco use during pregnancy is associated with lower hemoglobin levels; however, further exploration and clarification of this association need to be done for the wellness of public health. Our study's findings are consistent with the concept that tobacco exposure in any form during pregnancy has a negative impact on the pregnancy, newborn, and birth process. Confounding factors such as genetic predisposition that could not be excluded in the present study and previous obstetric history that was not properly recorded to look for factors that can affect present pregnancy outcomes can be regarded as the limitations of the current study. Further, we are planning to conduct a long-term longitudinal research plan on tobacco effects during pregnancy and their outcomes in neonates in a more expanded way in the state of Chhattisgarh where female tobacco users are more and research is unexplored in this direction.

## Conclusions

Our study findings indicated an adverse effect on their fetus and infants, as well as the pregnant females themselves who were exposed to tobacco. As per GATS 2016-2017, Chhattisgarh has a high percentage of tobacco users (39.1%), and among them, 24.6% were female tobacco users, which is highly significant. Policymakers need to ensure effective plans and strategies regarding tobacco use that pregnant females and their close relatives need to have enough knowledge about to avoid potential risks. Recently, the government and policymakers are using research-based data to create policies and guidelines for tobacco control in society, especially for pregnant females. These actions should consider the pertinent research findings from India for promoting tobacco control. Indian research activities in the fields of tobacco control and public health welfare must be reinforced to overcome such complications. Despite of high burden of tobacco use in Chhattisgarh, very few research have been done to show the impact of tobacco use among pregnant females and its outcome. A longitudinal research plan in Chhattisgarh state is needed to conclude long-term issues related to tobacco use during pregnancy to generate more data from such region of India where females are significantly using tobacco and tobacco-based products.
